# Raloxifene Suppresses Experimental Autoimmune Encephalomyelitis and NF-κB-Dependent CCL20 Expression in Reactive Astrocytes

**DOI:** 10.1371/journal.pone.0094320

**Published:** 2014-04-10

**Authors:** Rui Li, Wen Xu, Ying Chen, Wei Qiu, Yaqing Shu, Aimin Wu, Yongqiang Dai, Jian Bao, Zhengqi Lu, Xueqiang Hu

**Affiliations:** Multiple Sclerosis Center, Department of Neurology, The Third Affiliated Hospital of Sun Yat-Sen University, Guangzhou, Guangdong Province, China; Friedrich-Alexander University Erlangen, Germany

## Abstract

Recent clinical data have led to the consideration of sexual steroids as new potential therapeutic tools for multiple sclerosis. Selective estrogen receptor modulators can exhibit neuroprotective effects like estrogen, with fewer systemic estrogen side effects than estrogen, offering a more promising therapeutic modality for multiple sclerosis. The important role of astrocytes in a proinflammatory effect mediated by CCL20 signaling on inflammatory cells has been documented. Their potential contribution to selective estrogen receptor modulator-mediated protection is still unknown. Using a mouse model of chronic neuroinflammation, we report that raloxifene, a selective estrogen receptor modulator, alleviated experimental autoimmune encephalomyelitis–an animal model of multiple sclerosis–and decreased astrocytic production of CCL20. Enzyme-linked immunosorbent assay, immunohistochemistry imaging and transwell migration assays revealed that reactive astrocytes express CCL20, which promotes Th17 cell migration. In cultured rodent astrocytes, raloxifene inhibited IL-1β-induced CCL20 expression and chemotaxis ability for Th17 migration, whereas the estrogen receptor antagonist ICI 182,780 blocked this effect. Western blotting further indicated that raloxifene suppresses IL-1β-induced NF-κB activation (phosphorylation of p65) and translocation but does not affect phosphorylation of IκB. In conclusion, these data demonstrate that raloxifene provides robust neuroprotection against experimental autoimmune encephalomyelitis, partially via an inhibitory action on CCL20 expression and NF-κB pathways in reactive astrocytes. Our results contribute to a better understanding of the critical roles of raloxifene in treating experimental autoimmune encephalomyelitis and uncover reactive astrocytes as a new target for the inhibitory action of estrogen receptors on chemokine CCL20 expression.

## Introduction

Multiple sclerosis (MS) is a severe autoimmune disorder of the central nervous system (CNS) characterized by chronic inflammation, myelin loss, gliosis, varying degrees of axonal and oligodendrocyte pathology, and progressive neurological dysfunction [1]. Despite important advances in therapeutics for MS, none of the current disease-modifying drugs have been found to significantly alter the long-term prognosis of the disease. An increasing amount of clinical data indicates that estrogen (E2) may have therapeutic value for MS. Oral E2 administration exerts immunoregulatory effects and reduces the number and size of gadolinium-enhancing lesions in relapsing-remitting MS patients [2], [3]. Moreover, the disease usually shows increased relapse frequency and severity during the postpartum period [4], [5], whereas MS severity and relapse frequency decrease significantly during late pregnancy [6]. Newly developed synthetic selective estrogen receptor modulators (SERMs) that specifically target estrogenic mechanisms, but with fewer systemic estrogen side effects than E2, offer a promising therapeutic modality for the treatment of conditions associated with neuroinflammation [7]. However, the precise mechanism underlying the neuroprotection offered by SERMs in experimental autoimmune encephalopathy (EAE), an animal model of MS, remains to be elucidated. Determining where and how such medications act is of paramount importance to delineate the best sex steroid therapeutics for MS.

The actions of SERMs, similar to E2, are mediated primarily by estrogen receptors (ERs), especially ERα [8]–[10]. Although T lymphocytes, which play a major role in the pathology of MS and EAE, express ERs, bone marrow chimera experiments have indicated that ERα in inflammatory leukocytes may not be required for estrogen protection [11]. Recent studies have reported that signaling through ERα in astrocytes is essential for the beneficial effects of the ERα ligand in EAE [12], [13].

Astrocytes play an active role in the regulation of CNS autoimmunity [14]. Their harmful effects during chronic neuroinflammation have been demonstrated using mice with astrocyte-restricted knockout of upstream activators of NF-κB [15], which binds to the promoters of proinflammatory cytokine genes [16]. Among these, CCL20 is a highly regulated inflammatory chemokine that, via its receptor CCR6, drives recruitment of lymphocytes, especially Th17 cells–an important proinflammatory cell in EAE–to sites of CNS injury [17]–[20]. The important role of astrocytes in this proinflammatory effect through CCL20 signaling on inflammatory cells has been documented. CCR6 knockout mice are resistant to EAE induction [18]–[20], whereas specific neutralizing anti-CCL20 antibodies attenuate pathological changes in EAE [21]. However, their potential contribution to SERM-mediated protection is still unknown.

In the present study, we examined whether raloxifene (a SERM) treatment is able to reduce inflammation in EAE and CCL20 expression in reactive astrocytes. This is the first description of the effect of SERMs on CCL20 expression in astrocytes.

## Methods

### Ethics statements

All animal experiments were carried out according to a protocol approved by the Institutional Animal Ethical Committee of Jinan University (permission number: SYXK2012-0117). Surgery of mice was conducted after anesthetizing the animals with pentobarbital, and all efforts were made to minimize suffering.

### Mice

The ethical committee for animal experiments at Ji Nan University (Guangzhou, China) approved this study. Female C57BL/6 mice (Experimental Animal Center of Sun Yat-sen University, Guangzhou, China) were electronically tagged and kept, five animals per cage, under standard environmental conditions, and fed standard laboratory chow and tap water *ad libitum*.

### Ovariectomy (OVX) and raloxifene treatment

OVX and sham operation were performed at 6–8 weeks of age as described previously [22]. Ovaries were removed through a midline incision in the skin, and flank incisions in the peritoneum. The skin incision was then closed with metallic clips. Sham-operated animals had their ovaries exposed but not removed. Surgery was performed after the mice were anesthetized with pentobarbital. An OVX+Raloxifene group was given subcutaneous (s.c.) injections of raloxifene (SigmaAldrich, St Louis, MO, USA) (120 μg/mouse/day) dissolved in Miglyol 812 (OmyaPeralta GmbH, Hamburg, Germany). Sham+Vehicle and OVX+Vehicle groups received s.c. injections of Miglyol 812 (100 μl/mouse/day). Treatment with raloxifene or vehicle was started at the time of EAE induction, and continued until termination of the experiments.

### Induction of EAE and tissue collection

One week after surgery, the mice were immunized with myelin oligodendrocyte glycoprotein 35–55 peptide (MOG35-55), complete Freund's adjuvant (CFA), and pertussis toxin [23], [24]. Motor impairment was scored daily for clinical disease severity according to the 0–5 EAE grading scale [24]. At 19 days postimmunization (dpi) (peak), 6 mice in each group were randomly selected and deeply anesthetized. Spinal cords of mice were collected, placed in 4% paraformaldehyde and embedded in paraffin for histopathology and immunofluorescence analysis. Brain tissue was collected for flow cytometry. The other mice (n = 10 in each group) were observed for motor impairment until 30 dpi.

### Histopathology analysis

Spinal cord tissues embedded in paraffin were sectioned (4 μm thick) and stained with hematoxylin and eosin (HE) to reveal inflammatory infiltrates. Solochrome Cyanin technique was used for myelin staining. Pathological examination of spinal cords was performed in a blinded fashion. The scale evaluated for inflammation was as follows [25]: 0, no inflammatory cells; 1, a few scattered inflammatory cells; 2, organization of inflammatory infiltrates around blood vessels; and 3, extensive perivascular cuffing with extension into the adjacent parenchyma, or parenchymal infiltration without obvious cuffing. Demyelination in the spinal cords was scored as previously described [26], [27]: 1, traces of subpial demyelination; 2, marked subpial and perivascular demyelination; 3, confluent perivascular or subpial demyelination; 4, massive perivascular and subpial demyelination involving one half of the spinal cord with presence of cellular infiltrates in the CNS parenchyma; 5, extensive perivascular and subpial demyelination involving the whole cord section with presence of cellular infiltrates in the CNS parenchyma.

### Immunofluorescence

Spinal cord sections from EAE models (at 19 dpi, n = 6 in each group) were stained with neurofilament (NF) mouse mAb (Cell Signaling Technology, Beverly, MA, USA) followed by anti-mouse secondary antibody (Abcam (Hong Kong) Ltd, New Territories, HK) for evaluating axonal damage. Moreover, spinal cord sections and astrocytes cultured in vitro were dually stained with mouse anti-mouse GFAP (Millipore, Bedford, MA, USA) and rabbit anti-mouse CCL20 (Abcam (Hong Kong) Ltd, New Territories, HK), followed by incubation with anti-mouse and anti-rabbit secondary antibodies (Abcam (Hong Kong) Ltd, New Territories, HK). Stained sections were examined and photographed using a Leica DMI 4000B microscope (Leica Corp., Lasertechnik, Heidelberg, Germany) (for spinal cord sections) or Zeiss LSM 510 confocal microscope (Carl Zeiss, Jena, Germany) (for astrocytic cultures). Mean fluorescence intensity was calculated using Image Pro Plus (Media Cybernetics, Silver Spring, MD, USA).

### Flow cytometry analysis

CNS lymphocytes were collected as described previously [28] with some modifications. In brief, mice were perfused through the left ventricle with ice-cold 2 mM EDTA in PBS. The brain was dissected, cut into small pieces, and digested in collagenase D (1 mg/mL; Roche Diagnostics, Mannheim, Germany) and Dnase I (1 mg/ml; Sigma Aldrich, St Louis, MO, USA) for 45 min at 37 °C. Brain sections were passed through a 70-μm cell strainer, washed once in PBS, placed in a 30% Percoll solution, and pelleted for 20 min at 2000 rpm. CNS lymphocytes were isolated from the interface of a 30–70% discontinuous Percoll (Amersham Pharmacia Biotech, Piscataway, NJ, USA) gradient. Cells were resuspended in media and used for subsequent flow cytometry analysis. Cells were pre-incubated with Fc Block for 15 min at 4 °C in PBS containing 0.1% NaN3 and 2% BSA, followed by specific antibody staining. After stimulation with PMA (50 ng/ml, Sigma-Aldrich), ionomycin (1 μg/ml, Sigma-Aldrich) and BFA (3 μg/ml, Sigma-Aldrich) for 4 hours in a 37 °C incubator, the cells were then labeled with anti-CD4 FITC (eBioscience, San Diego, CA, USA) for cell surface staining, and anti-IL-17A PE (eBioscience) for cytoplasmic staining. Data collection was performed on FACS Calibur and LSR II flow cytometers using CELLQuest and FACs Diva software (Becton Dickinson, Franklin Lakes, NJ, USA) according to the setup recommendations of Maecker and Trotter [29]. Analysis was performed on a PC workstation using Flow Jo Software Ver. 7.6 (Trustees of Leland Stanford Jr. University, Tree Star, Inc).

### Primary astrocyte cultures and treatments

Astrocytes were cultured according to the protocol developed by McCarthy and deVellis [30] with some modifications. Neonatal C57BL/6 mice (1–2 days old; Experimental Animal Center of Sun Yat-sen University, Guangzhou, China) were decapitated, the hemispheres were removed, and the meninges discarded. After trypsin/DNase treatment, the tissues were dissociated mechanically and cells were passed through a cell strainer (70 μm). After centrifugation, the cells were resuspended in culture medium containing DMEM (F12 DMEM, Gibical), Glutamax, nonessential amino acids, 1 mM pyruvate sodium, 1% penicillin/streptomycin, and 10% fetal bovine serum (FBS) (Hyclone) and then plated in 75-cm^2^ flasks and incubated at 37 °C in a humid atmosphere with 5% CO2. The medium was changed twice weekly. When cultures became confluent, the flasks were shaken at 200 rpm for 12 h at 37 °C to dislodge microglia and oligoprecursors. The astrocyte-enriched layer was passaged into new flasks. When flasks became confluent again, cells were subcultured in poly-D-lysine-coated culture plates for experiments. The cultures consisted of >95% astrocytes as determined by immunoreactivity for GFAP. All experiments were carried out when the subcultures had become near to confluence. Astrocytes were switched to serum-free DMEM overnight before treatments. To study the kinetics of CCL20, the effects of IFN-γ (100 U/ml), TNF-α (20 ng/ml), and IL-1β (100 U/ml) were evaluated at 6, 12, 24 and 48 hours. Raloxifene and the ER antagonist ICI 182,780 were purchased from Sigma Aldrich. Recombinant cytokines were provided by PeproTech (Rocky Hill, NJ, USA). To evaluate effects of raloxifene on IL-1β-induced CCL20 expression on astrocytes, IL-1β (100 U/ml)-induced astrocytes were treated with raloxifene (10^−7^–10^−9^ M) or ICI 182, 780 (10^−7^ M) for 48 h.

### ELISA

An EAE model was established as described above. Supernatants of EAE brain tissue at different times in primary astrocyte culture were harvested. Measurement of cytokine levels in the supernatants of brain tissue and astrocyte cultures was performed by ELISA using commercially available ELISA kits, in accordance with the manufacturer's instructions. TNF-α and IL-1β ELISA kits were purchased from Bender (Coatesville, PA, USA). The CCL20 ELISA kit was obtained from R&D Systems (Minneapolis, MN, USA). Results are expressed as pg/ml.

### Th17 cell differentiation/polarization

Spleens of EAE mice were aseptically harvested at 19 dpi. Single-cell suspensions of splenocytes were prepared by pushing spleens through a sterile 70-μm pore size nylon mesh. CD4+CD62L+ T cells were isolated using MACS beads according to the manufacturer's protocol (Miltenyi Biotec, Bergisch Gladbach, Germany). CD4+CD62+ T cells were stimulated with plate-bound anti-CD3 antibodies (2 μg/ml; eBioscience) and soluble anti-CD28 antibodies (5 μg/ml; eBioscience) for 4 d in RPMI 1640 medium supplemented with 2 mM sodium pyruvate, L-glutamine, 10% FBS. T cells were polarized with human TGF-β (5 ng/ml; PeproTech), IL-6 (20 ng/ml; PeproTech), IL-23 (10 ng/ml; PeproTech) plus anti–IFN-γ (10 μg/ml; BD Biosciences, CA, USA) and anti-IL-4 (10 μg/ml; BD Biosciences) to stimulate Th17 differentiation.

### Transwell migration assay

Astrocytes were treated with IL-1β (100 U/ml) plus raloxifene (10^−7^–10^−9^ M) or ICI 182, 780 (10^−7^ M) for 48 h, and then the supernatants were collected and used as conditioned medium. Migration assays were performed using a 24-well transwell plate with a 3-μm pore size (Corning, NY, USA). A total of 4×10^5^ polarized Th17 cells were embedded in the upper chamber in serum-free RPMI 1640. Control or conditioned serum-free media were placed in the lower chamber. Cells were allowed to migrate for 4 h at 37°C. The cells that migrated to the lower chamber and were then stained for CD4 and IL-17, as described above, were counted by flow cytometry.

### Western blotting analysis

After each treatment, cells were rinsed twice with PBS, then nuclear protein was harvested using a Nuclear Extraction Kit (Pierce, Rockford, IL, USA) or total protein was obtained by M-PER Protein Extraction Buffer (Pierce) containing 1× protease inhibitor cocktail(Roche Diagnostics). Proteins were quantified using a BCA Kit (Pierce). Nuclear or total protein was separated by 10% sodium dodecyl sulphate-polyacrylamide gel electrophoresis (SDS-PAGE) and transferred onto a polyvinylidene difluoride (PVDF) membrane via semidry transfer. Membranes were blocked with 5% nonfat milk in Tris–phosphate buffer containing 0.05% Tween 20 (TBS-T). They were further incubated overnight at 4°C with primary antibodies including NF-κB p65, phospho-NF-κB (ser536), IκBα, phospho-IκBα (ser32) rabbit mAb, or rabbit anti-β-actin (all from Cell Signaling Technology, Beverly, MA, USA). The next day, horseradish peroxidase-conjugated secondary antibodies (Calbiochem, San Diego, CA, USA) were applied. Peroxidase-conjugated streptavidin and substrate were used for detection. The optical density of bands was evaluated using Image Pro Plus (Media Cybernetics, Silver Spring, MD, USA) and statistical comparisons were performed using GraphPad Prism version 5 software.

### Statistical analysis

Data in the figures are the results of three independent experiments, and are expressed as means ± SD. Differences between the means of experimental groups were analyzed using the two-tailed Student t test or one way ANOVA followed by Tukey's post hoc analysis (SPSS 16.0 program; SPSS, Chicago, IL, USA). *P*-values of <0.05 were regarded as statistically significant.

## Results

### OVX worsens EAE, while raloxifene treatment suppresses clinical signs and ameliorates pathological changes induced by OVX

**Figure 1:**
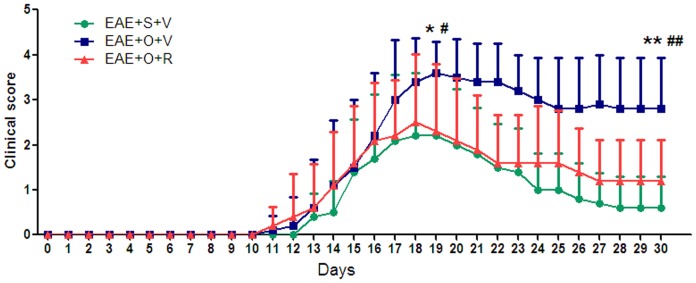
Motor impairment scores showing raloxifene treatment suppresses clinical signs of EAE following ovariectomy (n = 10 in each group). Subcutaneous injections (vehicle or raloxifene) were performed at the time of immunization. S: sham, V: vehicle, O: ovariectomy, R: raloxifene. *P<0.05 and **P<0.01 represent the statistical significance in two-tailed Student t test for the comparison between EAE+S+V and EAE+O+V groups; ^#^ *P*<0.05 and ^##^ *P*<0.01 represent the statistical significance in two-tailed Student t test for the comparison between EAE+O+V and EAE+O+R groups.

**Figure 2:**
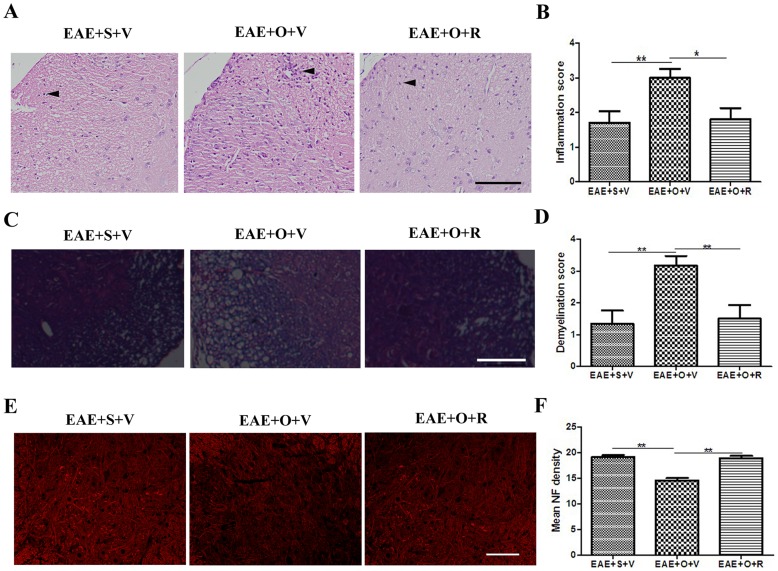
Raloxifene treatment ameliorates pathological changes of EAE following ovariectomy. A: HE staining showing that the amount of inflammatory cell infiltration (arrow) in the CNS at disease peak (at 19 dpi) in the EAE+S+V, EAE+O+V and EAE+O+R groups (Bar = 100 μm). B: The average scores for inflammation in the EAE+S+V, EAE+O+V and EAE+O+R groups (n = 6 in each group). C: Solochrome cyanin staining revealing demyelination in the EAE+S+V, EAE+O+V and EAE+O+R groups (Bar = 100 μm). D: The average scores for demyelination in the EAE+S+V, EAE+O+V and EAE+O+R groups (n = 6 in each group). E: Immunofluorescence of NF (showing axonal structures) in the EAE+S+V, EAE+O+V and EAE+O+R groups (Bar = 100 μm). F: Mean fluorescence intensity of NF in EAE+S+V, EAE+O+V and EAE+O+R groups (n = 6 in each group). S: sham, V: vehicle, O: ovariectomy, R: raloxifene. *P<0.05 and **P<0.01 represent the statistical significance in two-tailed Student t test.

Our study showed that the EAE+OVX+vehicle group had a higher motor impairment score respect to the EAE+sham+vehicle group (at 19 dpi: 3.6±0.7 *vs.* 2.2±1.4, *P* = 0.011; at 30 dpi: 2.8±1.1 *vs.* 0.6±0.7. *P*<0.001) (Figure 1), more severe inflammation (at 19 dpi: inflammation score: 3.0±0.8 *vs.* 1.7±1.1, *P* = 0.007) (Figure 2A–B), more severe demyelination (at 19 dpi: demyelination score: 3.2±0.8 *vs.* 1.3±1.0, *P* = 0.005) (Figure 2C–D) and more severe axonal damage (at 19 dpi: mean fluorescence intensity of NF: 14.6±1.0 *vs.* 19.2±0.9, *P*<0.001) (Figure 2E–F). Subcutaneous injection of raloxifene in C57BL/6 mice immunized with MOG35–55 peptide decreased the motor impairment score at 19 dpi (2.3±1.5 *vs.* 3.6±0.7, *P* = 0.027) and the score further declined at 30 dpi (1.2±0.9 *vs.* 2.8±1.1, *P* = 0.003) (Figure 1). Moreover, raloxifene protected against inflammatory cells homing to the CNS, demyelination, and axonal damage in castrated EAE model animals (inflammation score in EAE+OVX+raloxifene group and EAE+OVX+vehicle group at 19 dpi: 1.8±1.0 *vs.* 3.0±0.8, *P* = 0.01; demyelization score at 19 dpi: 1.5±1.0 *vs.* 3.2±0.8, *P* = 0.009; mean fluorescence intensity of NF: 19.0±1.0 *vs.* 14.6±1.0, *P*<0.001) (Figure 2A–F).

OVX increases astrogliosis, CCL20 expression and Th17 cell infiltration in the CNS, and raloxifene treatment alleviates such astrogliosis, CCL20 expression and Th17 cell infiltration.

**Figure 3:**
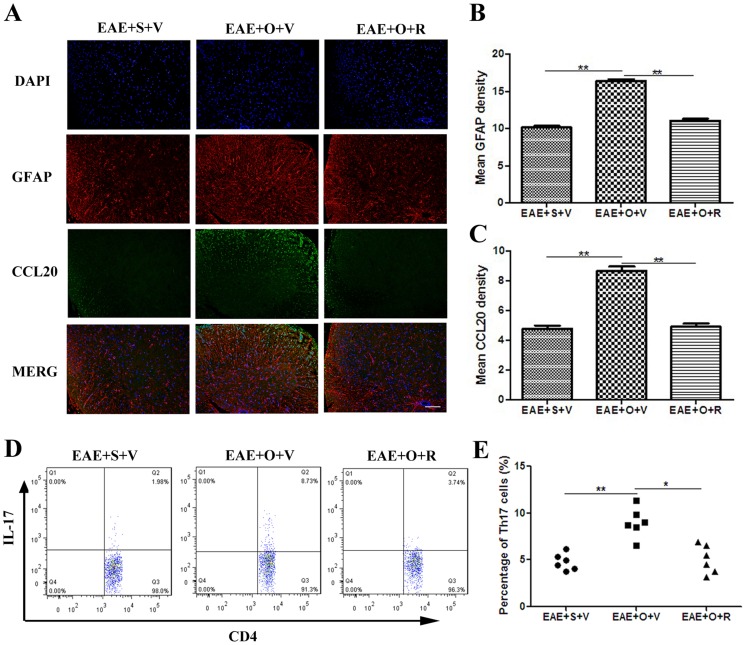
OVX increases astrogliosis, CCL20 expression and Th17 cell infiltration in the CNS, and raloxifene treatment alleviates such effects by OVX. A: Immunofluorescence of GFAP (showing astrogliosis) and CCL20 in the EAE+S+V, EAE+O+V and EAE+O+R groups (Bar = 100 μm). B: Mean fluorescence intensity of GFAP in the EAE+S+V, EAE+O+V and EAE+O+R groups (n = 6 in each group). C: Mean fluorescence intensity of CCL20 in EAE+S+V, EAE+O+V and EAE+O+R groups (n = 6 in each group). D: The percentage of infiltrating Th17 cell among CD4+T cells in the CNS (at 19 dpi) in the EAE+S+V, EAE+O+V and EAE+O+R groups. E: Comparison of the average percentages of infiltrating Th17 cell in the CNS in the EAE+S+V, EAE+O+V and EAE+O+R groups (n = 6 in each group). S: sham, V: vehicle, O: ovariectomy, R: raloxifene. dpi: days postimmunization. *P<0.05 and **P<0.01 represent the statistical significance in two-tailed Student t test.

Immunofluorescence analysis showed that the mean fluorescence intensity of GFAP and CCL20 were higher in EAE+OVX+vehicle group than EAE+sham+vehicle group (GFAP: 16.4±0.7 vs. 10.1±0.6, *P*<0.001; CCL20: 8.7±0.7 vs. 4.8±0.5, P<0.001), and raloxifene reversed such effect of OVX (GFAP: 11.1±0.6 *vs.* 16.4±0.7, *P*<0.001; CCL20: 4.9±0.6 *vs.* 8.7±0.7, *P*<0.001) (Figure 3A–C). Flow cytometry analysis showed that OVX increased the percentage of Th17 cell infiltration in the CNS at 19 dpi (EAE+OVX+vehicle *vs.* EAE+sham+vehicle: (9.0±1.8)% *vs.* (4.1±1.3)%, *P*<0.001), and that raloxifene reversed this pathologic process (EAE+OVX+raloxifene *vs.* EAE+OVX+vehicle: (5.1±1.5)% *vs.* (9.0±1.8)%, *P* = 0.001)(Figure 3 D–E).

Astrocytes express CCL20 in vitro and vivo.

**Figure 4:**
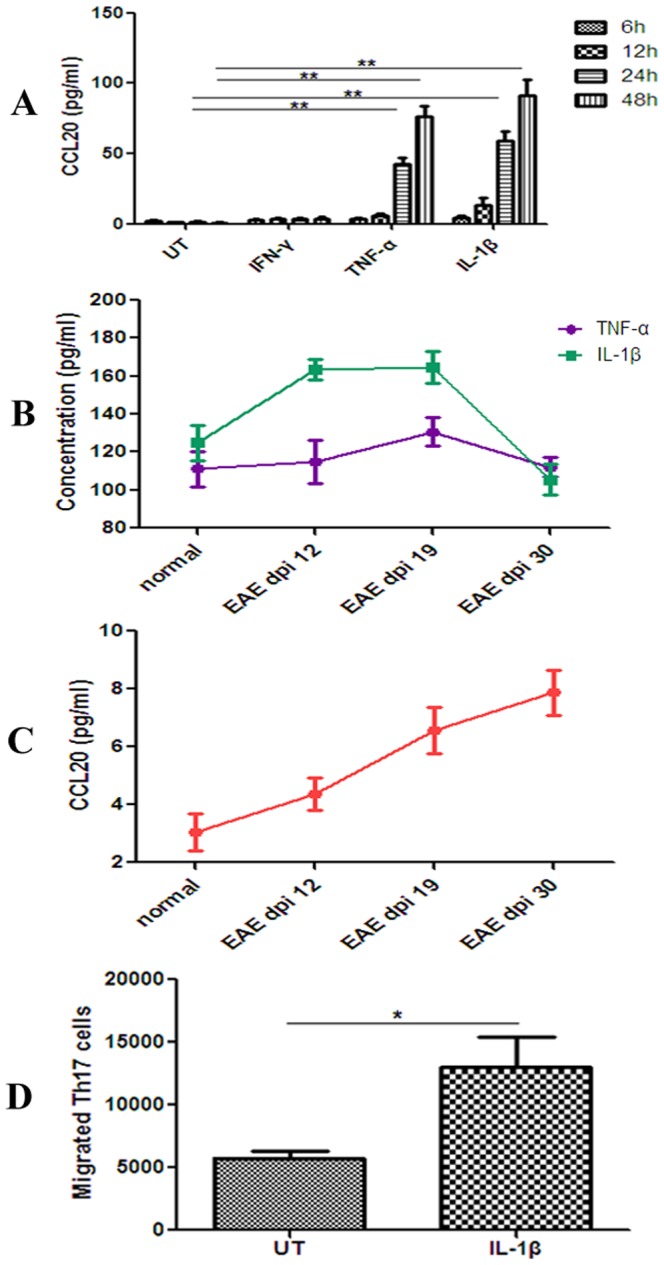
Astrocytes express CCL20 and astrocyte-derived CCL20 promotes Th17 cell migration. A: The expression of CCL20 in astrocytes is strongly induced by IL-1β (100 U/mL) and TNF-α (20 ng/ml) in vitro (n = 3 in each group). B–C: Increased TNF-α and IL-1β concentrations followed by elevated CCL20 levels in the brain tissue supernatants of EAE mice (n = 3 in each group). D: Th17 cell migration was increased in IL-1β (100 U/mL)-stimulated astrocyte conditioned media, compared with control media (n = 3 in each group). dpi: days postimmunization; UT: untreated. *P<0.05 and **P<0.01 represent the statistical significance in two-tailed Student t test.

We analyzed CCL20 expression in astrocytes both in vitro and in vivo. By ELISA, CCL20 expression in astrocytes in vitro was either undetectable or it was expressed at very low levels in unstimulated astrocytes. TNF-α (20 ng/ml) and IL-1β (100 U/ml), but not IFN-γ (100 U/ml) steadily induced CCL20 production in astrocytes. CCL20 production was maximal following IL-1β (100 U/ml) stimulation for 48 hours (Figure 4A). In vivo, immunofluorescence also detected immuoreactivity to CCL20 mainly expressed in astrocytes in EAE mice (Figure 3A). Increased TNF-α and IL-1β concentrations followed by elevated CCL20 levels were found in the brain tissue supernatants of EAE mice (Figure 4B–C).

Astrocyte-derived CCL20 promotes Th17 cell migration in vitro.

CD4+CD62L+ T cells were cultured under Th17 polarizing conditions. Cells cultured under Th17 polarizing conditions express high levels of CCR6 and were used in transwell migration assays. Th17 cell migration was increased in IL-1β astrocyte conditioned media, compared with control media (13,033±5873 *vs.* 5756±1440, *P* = 0.028) (Figure 4D).

Raloxifene decreases astrocytic CCL20 expression and Th17 cells migration in vitro.

**Figure 5:**
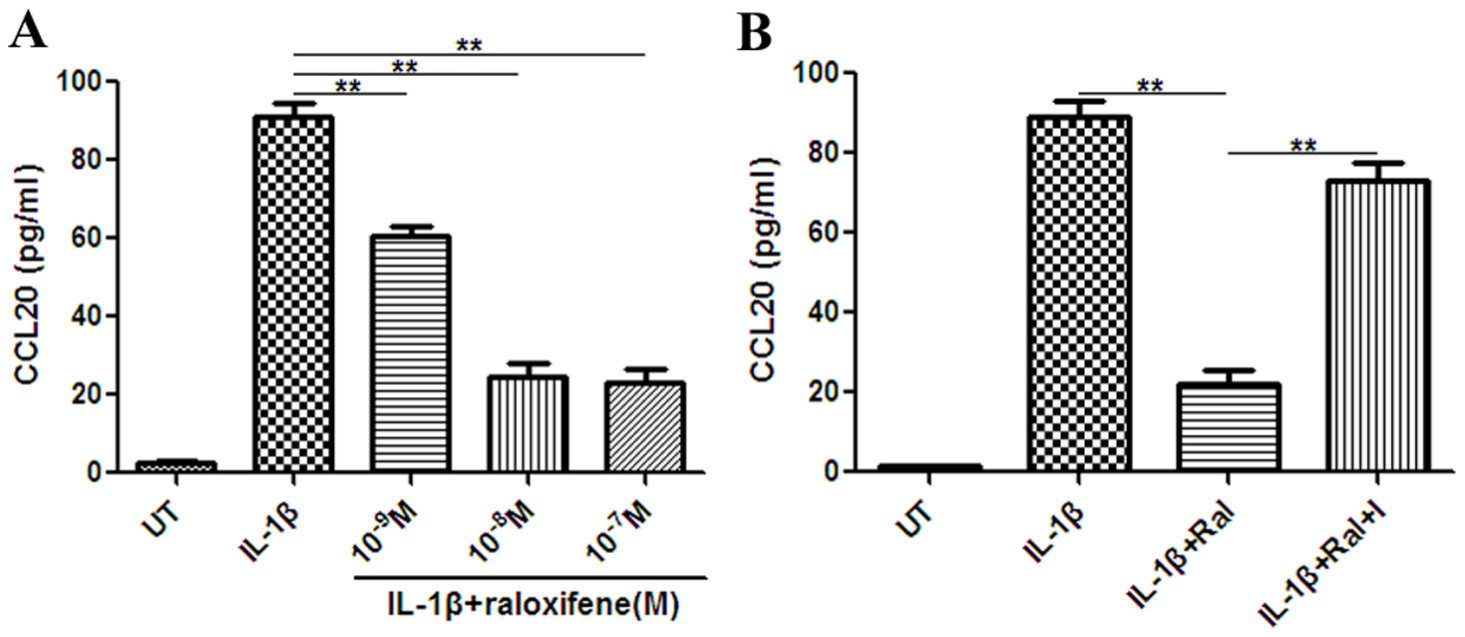
Effect of raloxifene on astrocytic CCL20 production. A: Raloxifene at 10^−9^–10^−7^ M significantly reduced the expression of CCL20 in astrocytes (n = 3 in each group). B: ICI 182, 780 (10^−7^ M) eliminated the effect of raloxifene at 10^−8^ M on astrocytic CCL20 expression (n = 3 in each group). UT: untreated, Ral: raloxifene, I: ICI 182, 780. **P<0.01 represent the statistical significance in two-tailed Student t test.

**Figure 6:**
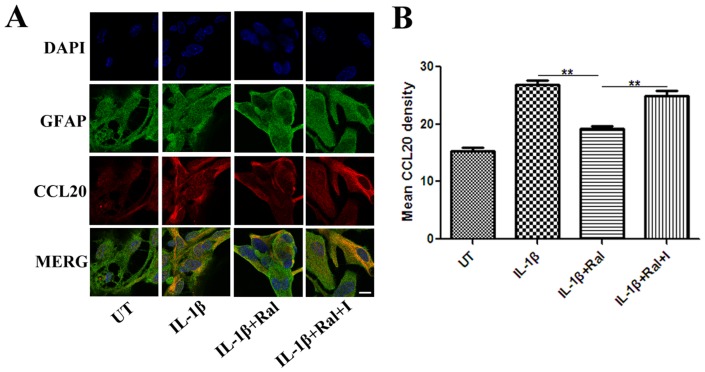
A: Immunofluorescence staining for GFAP and CCL20 in astrocytes with different treatments (Bar = 10 μm). B: Semi-quantitative measurement of mean fluorescence intensity showed that mean fluorescence intensity of IL-1β (100 U/ml)-induced CCL20 expression was lower in the IL-1β+raloxifene (10^−8^ M) treatment group than in the IL-1β treatment group, while ICI 182, 780 (10^−7^ M) reversed the effect of raloxifene at 10^−8^ M on CCL20 expression (n = 3 in each group). UT: untreated, I: ICI 182,780, Ral: raloxifene. **P<0.01 represent the statistical significance in two-tailed Student t test.

Raloxifene treatment at different concentrations from 10^−9^ to 10^−7^ M for 48 h significantly decreased IL-1β (100 U/ml)-induced astrocytic CCL20 expression, as assessed by ELISA (Figure 5A). IL-1β (100 U/ml) + Raloxifene (10^−8^ M) - treated astrocytes produced lower level of CCL20 than IL-1β (100 U/ml)-treated astrocytes (IL-1β+Ral *vs.* IL-1β: (22.0±8.6) pg/ml *vs.* (88.9±10.3) pg/ml, *P*<0.001).The ER antagonist ICI 182,780 (10^−7^ M) eliminated the effect of raloxifene at 10^−8^ M on astrocytic CCL20 expression (IL-1β+Ral+I *vs.* IL-1β+Ral: (72.9±11.2) pg/ml *vs.* (22.0±8.6) pg/ml, *P*<0.001) (Figure 5B). We also performed immunofluorescence staining for GFAP and CCL20 in astrocytes with different treatments. Semi-quantitative measurement of mean fluorescence intensity showed that the mean fluorescence intensity of IL-1β-induced CCL20 expression was lower in the IL-1β+raloxifene treatment group than in IL-1β treatment group (IL-1β+Ral *vs.* IL-1β: (19.2±0.8) *vs.* (26.9±1.2), *P* = 0.001). ICI 182, 780 (10^−7^ M) reversed the effect of raloxifene at 10^−8^ M on CCL20 expression (IL-1β+Ral+I *vs.* IL-1β+Ral: (24.9±1.7) *vs.* (19.2±0.8), *P* = 0.006) (Figure 6A–B).

**Figure 7:**
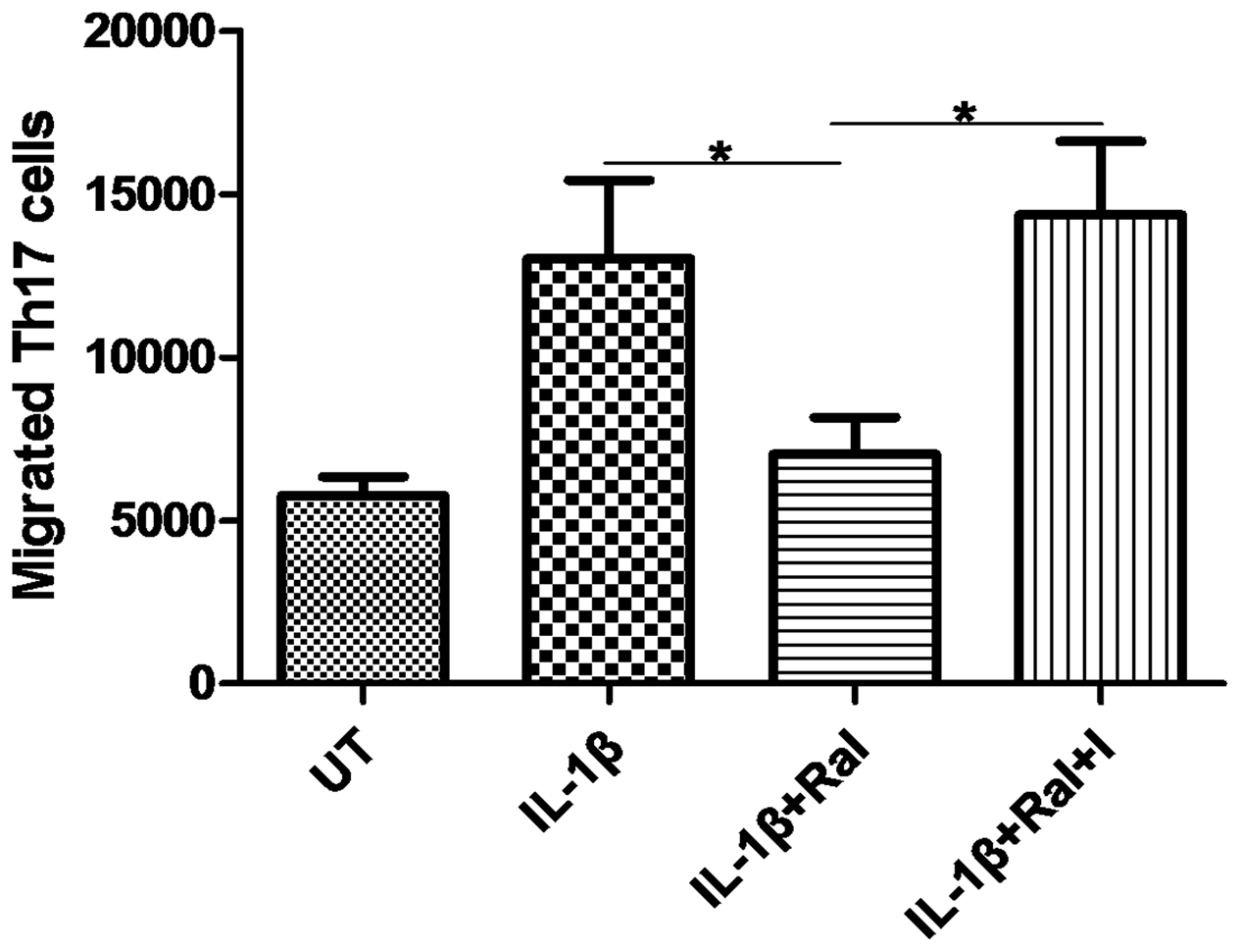
Th17 cell transwell migration assay in control and different conditioned media. Th17 cell migration decreased significantly in raloxifene (10^−8^ M)-treated astrocyte conditioned media, compared with IL-1β (100 U/ml)-treated astrocyte conditioned media (n = 3 in each group). UT: untreated, I:ICI 182,780, Ral: raloxifene. *P<0.05 represent the statistical significance in two-tailed Student t test.

Furthermore, raloxifene inhibits astrocyte-derived CCL20-mediated Th17 cell migration in vitro. Th17 cell migration was markedly decreased in IL-1β plus raloxifene-conditioned media (IL-1β+Ral *vs.* IL-1β: 7029±2760 *vs.* 13033±5873, *P* = 0.047), and this effect was inhibited by ICI 182, 780 (IL-1β+Ral+I *vs.* IL-1β+Ral: 14366±5492 *vs.* 7029±2760, *P* = 0.015) (Figure 7).

Raloxifene attenuates nuclear transcription factor NF-κB activation and translocation in cultured astrocytes.

**Figure 8:**
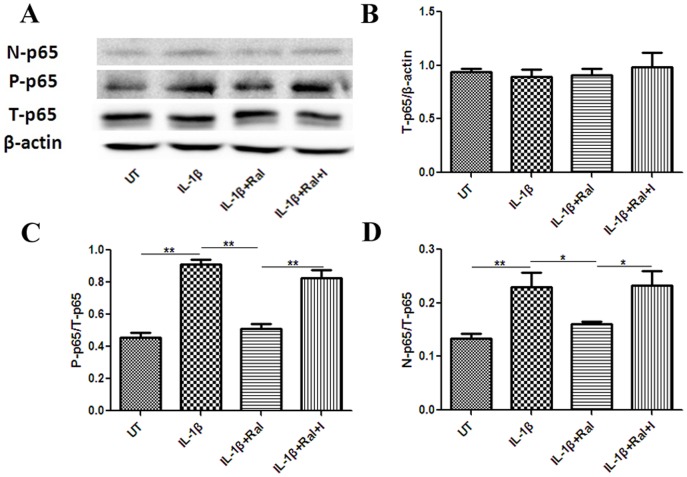
Raloxifene attenuates nuclear transcription factor NF-κB activation and translocation in cultured astrocytes. Raloxifene (10^−8^ M) did not impede T-p65 amount(A and B), but it inhibited P-p65 (A and C) and N-p65 amount(A and D) in IL-1β (100 U/mL)-stimulated astrocytes (n = 3 in each group). ICI 182,780 (10^−7^ M) reversed this effect of raloxifene. N-p65: nuclear p65, P-p65: phosphor-p65, T-p65: total p65, UT: untreated, I:ICI 182,780, Ral: raloxifene. *P<0.05 and **P<0.01 represent the statistical significance in one way ANOVA followed by Tukey's post hoc analysis.

**Figure 9:**
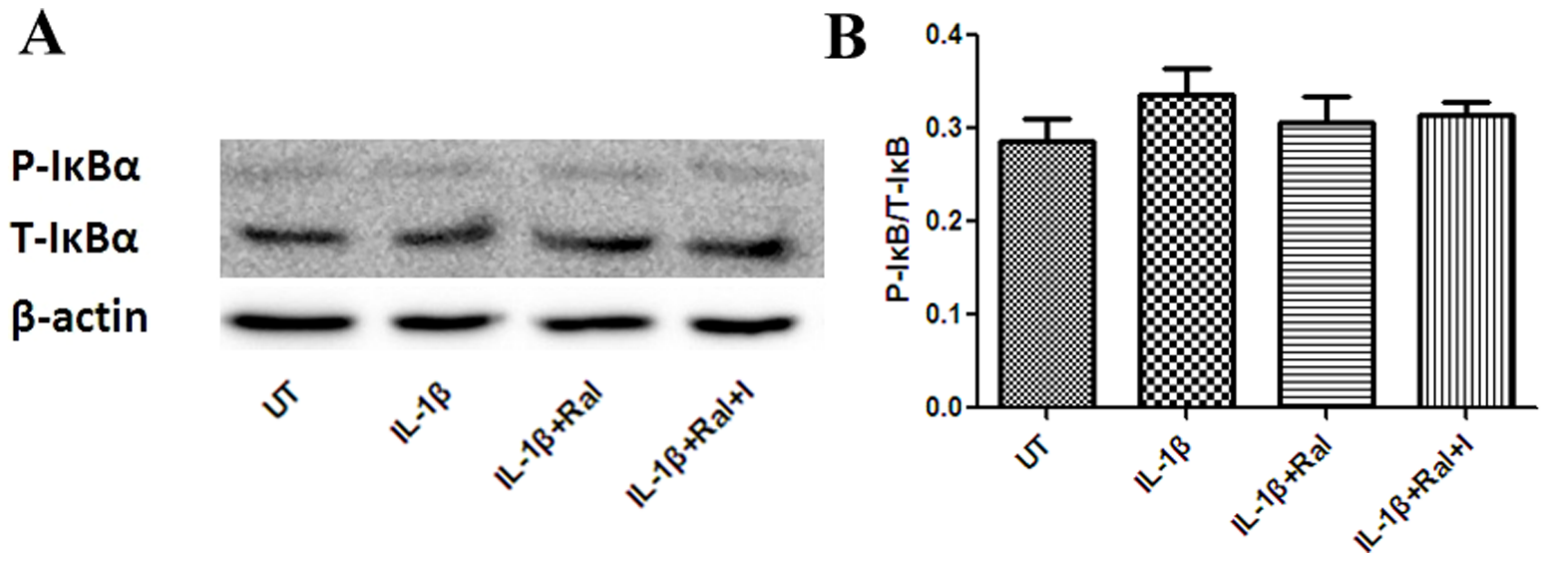
Raloxifene (10^−8^ M) did not affect the phosphorylation status of IκBα in IL-1β (100 U/mL)-induced astrocytes (n = 3 in each group). P-IκBα: phosphor-IκBα, UT: untreated, I: ICI 182,780, Ral: raloxifene.

Levels of total p65 (NFκB subunit) (T-p65), nuclear p65 (N-p65) and phosphorylated p65(P-p65) were analyzed by western blotting. Raloxifene did not affect T-p65 expression, but it inhibited N-p65 and P-p65 expression in IL-1β (100 U/mL)-stimulated astrocytes, which suggests that raloxifene attenuates p65 phosphorylation and translocation (Figure 8A–D). Total IκBα (T-IκBα), and phosphorylated IκBα (P-IκBα) expression were also analyzed. We found that raloxifene did not affect phosphorylation of IκBα in IL-1β (100 U/mL)-induced astrocytes (Figure 9A–B).

## Discussion

In this study, we examined the therapeutic effects of raloxifene (s.c. injection) on chronic EAE at the time of immunization in C57BL/6 mice with a special focus on astrocyte reactivity in vivo and in vitro.

Recent clinical data have led us to consider sexual steroids as new potential therapeutic tools for MS [31]. Exploring the cellular mechanism underlying the neuroprotection offered by SERMs is helpful for further research on neuro-SERMs that specifically target estrogenic mechanisms of neuroprotection, but with fewer systemic estrogen side effects. Our data revealed a drastic reduction in EAE motor impairment scores, inflammatory infiltrates, demyelination, astrogliosis and axonal damage following raloxifene supplementation, supporting the notion that SERMs can alleviate ongoing chronic neuroinflammation [7], [32], [33] and subsequent pathological process. Th17 cells play a critical role in EAE development [34], and the recruitment of Th17 cells depends on the CCL20/CCR6 axis [19], [35]. Our study provides additional evidence that raloxifene reduces CCL20 expression and Th17 cell infiltration in CNS of EAE.

Our in vitro and in vivo experimental evidence confirmed that the main source of CCL20 in the CNS of EAE is TNF-α or IL-1β activated astrocytes [17],[35]. In vitro, the present data show that IL-1β and TNF-α strongly induce CCL20 production by cultured astrocytes. Astrocyte-derived CCL20 can then promote migration of Th17 cells. In vivo, we observed that the distribution of CCL20 expression was mainly in accordance with the location of GFAP-positive cells, and that the level of CCL20 in brain tissue supernatants increased (at the peak and the late in the disease course in EAE model animals) subsequent to the elevation of TNF-α and IL-1β levels. This finding led us to speculate that microglia, which are activated earlier than astrocytes, promote astrocytic activation though IL-1β and TNF-α derived predominantly from microglia [17], [36]–[38]. Then, astrocyte-derived CCL20 further promotes Th17 cell migration into the inflamed sites in EAE model animals and exacerbates the disease at its peak and late in the disease course.

Since raloxifene reduces Th17 cell infiltration and CCL20 expression (mainly by astrocytes) in CNS of EAE, we further determined if such inhibition of Th17 cell infiltration is due to the suppressive effect of raloxifene on CCL20 expression in astrocytes. We found that raloxifene treatment at 10^−8^ M for 48 h decreased IL-1β (100 U/ml)-induced astrocytic CCL20 expression and subsequent Th17 cell migration which suggests that the decrease in the number of Th17 cells homing to the CNS is related to the suppression of astrocytic CCL20 production owing to raloxifene. The finding that ICI 182, 780 can reverse the effect of raloxifene corroborates the involvement of astrocytic ERs in this anti-inflammation process.

We further investigated the upstream mechanism by which raloxifene inhibits astrocytic CCL20 production. CCL20 expression is highly regulated by NF-κB [39]. NF-κB is an important factor in proinflammatory signaling, regulating both effector and target genes. Moreover, NF-κB can interact with ER pathways [16]. ERα gene transfer inhibits NF-κB activation in vascular smooth muscle (VSM) cells from female rats treated with IL-1β [40]. One recent study showed that the action of SERMs such as ospemifene and bazedoxifene on astrocytes involves the blockade of translocation of p65 to the nucleus and the consequent inhibition of NF-κB-induced transcription of pro-inflammatory chemokines and cytokines [41]. Thus, we tested whether raloxifene exhibits a similar inhibition of NF-κB activity, resulting in decreased CCL20 expression in astrocytes. In our study, raloxifene inhibited N-p65 and P-p65 expression in IL-1β (100 U/mL)-stimulated astrocytes, which suggests that the anti-inflammatory action of raloxifene toward CCL20 astrocytic production could result from molecular mechanisms involving modulation of NF-κB signaling via inhibition of p65 translocation and activation.

NF-κB is held inactive in the cytoplasm by inhibitory IκB proteins. The degradation of IκB unmasks the nuclear localization signal of the NF-κB/Rel family protein, leading to its nuclear translocation and binding to enhancers or promoters of target genes [42]. Therefore we next examined whether raloxifene inhibits the phosphorylation and translocation of p65 by decreasing IκB phosphorylation and degradation. However, raloxifene seems not to inhibit the translocation and phosphorylation of p65 via reducing degradation of IκB as neither total nor phosphorylated IκB expression was affected by raloxifene treatment. NF-κB activation can be uncoupled functionally from IκB phosphorylation and degradation [42]. PI3K and Akt are also necessary for p65 phosphorylation induced by IL-1β and activated PI3K and Akt appear to induce NF-κB-dependent transcription by activating p65 rather than by promoting the degradation of IκB [42]. Furthermore, in cells such as microglia/macrophages, the anti-inflammatory effects of E2 involve ERα-mediated activation of PI3K, preventing nuclear translocation of NF-κB [43]. We therefore presume that raloxifene inhibits NF-κB activation by suppressing PI3K and Akt. Further study of the effect of raloxifene on PI3K and Akt activation in astrocytes may answer this question.

In conclusion, these data demonstrate that raloxifene provides robust neuroprotection against EAE, partially via an inhibitory action on CCL20 expression and NF-κB pathways in reactive astrocytes. Our results contribute to a better understanding of the critical roles of raloxifene in treating EAE and uncover reactive astrocytes as a new target for the inhibitory action of ERs on chemokine CCL20 expression.
